# Evolution of severe sleep-wake cycle disturbances following traumatic brain injury: a case study in both acute and subacute phases post-injury

**DOI:** 10.1186/s12883-016-0709-x

**Published:** 2016-09-27

**Authors:** Catherine Duclos, Marie Dumont, Marie-Julie Potvin, Alex Desautels, Danielle Gilbert, David K Menon, Francis Bernard, Nadia Gosselin

**Affiliations:** 1 Center for Advanced Research in Sleep Medicine, Hôpital du Sacré-Coeur de Montréal, 5400 boul. Gouin Ouest, local E-0300, Montréal, Québec H4J 1C5 Canada; 2 Department of Psychiatry, Université de Montréal, Montréal, Canada; 3 Traumatology program, Hôpital du Sacré-Coeur de Montréal, 5400 boul. Gouin Ouest, Montréal, Québec H4J 1C5 Canada; 4 Department of Neuroscience, Université de Montreal, Montréal, Canada; 5 Division of Anaesthesia, University of Cambridge, Addenbrooke’s Hospital, Box 93, Cambridge, CB2 2QQ UK; 6 Department of Medicine, Université de Montreal, Montréal, Canada; 7 Department of Psychology, Université de Montréal, Montréal, Canada; 8 Department of Radiology, Hôpital du Sacré-Cœur de Montréal, 5400 boul. Gouin Ouest, local E-0330, Montréal, Québec H4J 1C5 Canada

**Keywords:** Traumatic brain injury, Sleep disorders, Actigraphy, Circadian rhythms, Neurocritical care, Neuropsychiatry

## Abstract

**Background:**

Sleep-wake disturbances are frequently reported following traumatic brain injury (TBI), but they remain poorly documented in the acute stage of injury. Little is known about their origin and evolution.

**Case presentation:**

This study presents the case of a patient in the acute phase of a severe TBI. The patient was injured at work when falling 12 m into a mine and was hospitalized in the regular wards of a level I trauma centre. From days 31 to 45 post-injury, once he had reached a level of medical stability and continuous analgosedation had been ceased, his sleep-wake cycle was monitored using actigraphy. Results showed significant sleep-wake disturbances and severe sleep deprivation. Indeed, the patient had an average nighttime sleep efficiency of 32.7 ± 15.4 %, and only an average of 4.8 ± 1.3 h of sleep per 24-h period. After hospital discharge to the rehabilitation centre, where he remained for 5 days, the patient was readmitted to the same neurological unit for paranoid delusions. During his second hospital stay, actigraphy recordings resumed from days 69 to 75 post-injury. A major improvement in his sleep-wake cycle was observed during this second stay, with an average nighttime sleep efficiency of 96.3 ± 0.9 % and an average of 14.1 ± 0.9 h of sleep per 24-h period.

**Conclusion:**

This study is the first to extensively document sleep-wake disturbances in both the acute and subacute phases of severe TBI. Results show that prolonged sleep deprivation can be observed after TBI, and suggest that the hospital environment only partially contributes to sleep-wake disturbances. Continuous actigraphic monitoring may prove to be a useful clinical tool in the monitoring of patients hospitalized after severe TBI in order to detect severe sleep deprivation requiring intervention. The direct impact of sleep-wake disturbances on physiological and cognitive recovery is not well understood within this population, but is worth investigating and improving.

**Electronic supplementary material:**

The online version of this article (doi:10.1186/s12883-016-0709-x) contains supplementary material, which is available to authorized users.

## Background

Chronic sleep-wake disturbances, such as insomnia and hypersomnia, are among the most widely-reported sequelea following traumatic brain injury (TBI), and have been documented across all levels of TBI severity, until several years post-injury [1]. Less attention has been paid to sleep-wake disturbances that occur in the first weeks post-injury. This might be explained by the challenges of performing sleep studies in an acute care setting, where most patients are confused and are not able to evaluate their own sleep quality.

A first group of studies that aimed at documenting sleep disturbances in post-acute TBI used nurse observations in individuals admitted to rehabilitation centres. One study found that of 31 patients, 21 (68 %) had two or more hours awake during the night [2]. Similarly, a second study showed that mild to severe sleep disturbances were present among 84 % of TBI patients upon rehabilitation admission, and persisted for 66 % of patients one month post-injury [3]. This research group used item one of the Delirium Rating Scale-Revised-98 to classify the severity of sleep-wake cycle disturbances as none, mild, moderate, or severe.

With the aim of using more objective methods to document the sleep-wake cycle of patients in the acute and post-acute phases of moderate-severe TBI, a second group of studies used actigraphy, which measures physical motion over time, to derive a rest-activity pattern. It has been shown that the rest-activity cycle measured with actigraphy strongly correlates with the sleep-wake cycle [4]; consequently, the rest-activity cycle derived from actigraphy is often referred to as the sleep-wake cycle. Within this context, a study carried out during early rehabilitation found that 11 of 14 moderate-severe TBI patients had an average sleep efficiency lower than 63 %, pointing to pervasive sleep-wake disturbances [5]. More recently, Gardani and colleagues evaluated 30 patients with chronic severe TBI in an inpatient rehabilitation setting, using actigraphy and self-report measures [6]. The authors found that 67 % of patients had sleep-wake cycle disturbances, 50 % of which met diagnostic criteria for a sleep disorder. Additionally, we recently used 10-day actigraphy recordings with 16 TBI patients hospitalized in a level I trauma centre in order to quantify the clustering of activity during the daytime and of rest during the nighttime as an estimate of their sleep-wake cycle consolidation. We found that patients had a poor sleep-wake cycle consolidation, which gradually improved over time [7]. However, using a threshold of ≥80 % of all 24-h activity occurring in the daytime, only half of the patients reached an acceptable sleep-wake cycle consolidation during the recording period. Patients who reached an acceptable sleep-wake cycle consolidation were more likely to emerge from posttraumatic amnesia (PTA) and to have lower disability at hospital discharge.

Despite the high prevalence of acute and subacute sleep-wake disturbances in TBI patients, their aetiology is not well understood. Furthermore, no study has yet documented the sleep-wake cycle during both the acute and subacute phases of TBI, while the patient was hospitalized in the same environment, which is of interest given that the hospital environment itself may be a contributing factor to disturbed sleep and wake. The aim of this article is to document the case of one of our TBI patients from the abovementioned study [7], who suffered severe sleep-wake cycle disturbances during his acute hospital stay. Since this patient was readmitted 5 days post-discharge and wore the actigraph during his second hospital stay, his case enables us to document the evolution of his sleep-wake cycle over time and to juxtapose the sleep-wake cycle recorded during two different hospital stays in a similar environment, during the acute (measured days 31–45 days post-injury, starting 4 days following discharge from the intensive care unit (ICU)) and subacute (measured days 69–75 post-injury) phases.

## Case presentation

### Biographical history

LC is a 43-year-old right-handed Caucasian male, who resides with his spouse and two teenage daughters. Prior to his injury, LC was in good physical health, had no previous history of TBI, chronic disease, drug or alcohol abuse, or psychiatric, neurological or sleep disorders.

### Injury

LC suffered a severe TBI when falling 12 m into a well of a mine during work hours. LC lost consciousness, had an initial Glasgow Coma Scale (GCS) score of 6 [8], and was immediately transported by ambulance to the nearest hospital, located approximately 160 km from the site of injury. Upon arrival at the regional hospital, his GCS score was 8. Following clinical evaluation, he was immediately transferred by ambulance to a level I trauma centre located over 500 km from the site of injury. A level I trauma centre provides the highest level of surgical and specialized care to trauma patients, is comprised of a full range of equipment and specialists dedicated to the care of patients having suffered TBI or orthopaedic injuries, and generally receives the most severe cases within a large geographical area.

#### First admission

**Fig. 1:**
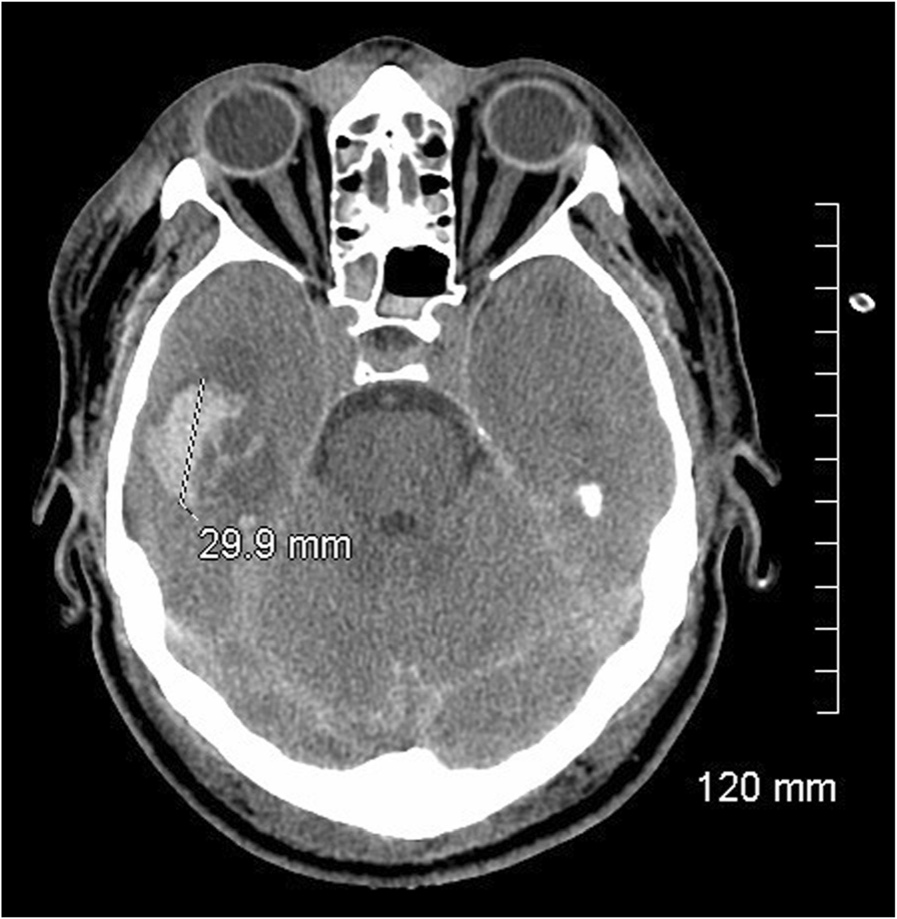
CT scan at admission. Initial CT scan taken at admission, showing right temporal parenchymal hematoma, diffuse subarachnoid haemorrhage in the left hemisphere, and diffuse axonal injury

LC was admitted to the trauma centre approximately 15 h after injury. His GCS score was three (intubated) upon admission to the emergency room, and he was taken to the ICU. A computed tomography (CT) scan revealed diffuse subarachnoid haemorrhage in the left hemisphere, left parieto-occipital subdural hematoma, right temporal intraparenchymal hematoma (3 cm), intrapeduncular, left intrapontine and temporal petechiae, as well as left frontal and right parieto-occipital contusions (see Fig. 1). His Marshall score was two [9], and his Rotterdam score was also two [10]. LC also suffered multiple facial fractures, a C4 cervical fracture, D6, D8 and D12 thoracic fractures, a fracture of the left 9^th^ rib, a spleen laceration, a pseudo-aneurysm of the aorta (4 mm), and a left pneumothorax.

LC was hospitalized in the ICU for 27 days. Overall, he was under continuous sedation for 16 days, during which time he received an average daily dose of 4.79 ± 2.33 g of propofol, and 6.2 ± 2.2 mg of fentanyl. During 11 of those 16 days of continuous sedation, LC also received an average daily dose of 0.55 ± 0.25 mg of midazolam. He was intubated 25 days, had elevated intracranial pressure (≥20 mmHg) during 13 days with a peak at 46.3 ± 14 mmHg, and had on average 7.3 ± 14 episodes of elevated intracranial pressure per day.

LC began responding to simple orders 15 days post-injury, when sedation was interrupted briefly to assess his level of response, and he opened his eyes 16 days post-injury. Subsequent to ICU discharge (27 days post-injury), LC was transferred to a six-patient room in the neurological ward. LC suffered akinetic mutism and moderate-severe oropharyngeal dysphagia throughout the first 46 days post-injury, and he then began to whisper, reaching a normal voice level 2 days prior to hospital discharge. At this point, he could walk unassisted and was fully functional in all bed and chair transfers.

LC was discharged from the trauma centre 55 days post-injury and admitted to a 200-bed inpatient rehabilitation centre, specialized in the care of TBI, orthopaedic injuries and neurology. Within the 72 h prior to hospital discharge, LC had a score of 10 out of 29 on the Disability Rating Scale [11], reflecting confused communication ability, partial cognitive disability for grooming, a markedly dependent level of functioning (mental, emotional, or social), and a non-competitive level of employability. The neurological examination carried out 8 days prior to hospital discharge (47 days post-injury) yielded a score of 5 on the Neurological Outcome Scale for Traumatic Brain Injury without the supplemental items [12]. Deficits arose when LC was asked the current month and his age, which he both answered incorrectly, as well as when asked to identify odours or name objects for stimulus cards. This could either be the result of mild to moderate aphasia, or PTA, which would account for an inability to recall the words associated to various stimuli. Due to persistent akinetic mutism throughout most of the hospitalization period, neuropsychological evaluations were only carried out during the second hospital stay.

#### Second admission

Five days after his admission to the inpatient rehabilitation centre, LC was readmitted to the trauma centre by ambulance for persecutory paranoid delusion, as per clinical observations at the inpatient rehabilitation centre, and remained hospitalized for 43 days in a two-patient room of the same neurological ward on which he had previously been hospitalized.

During this second hospital stay, LC continuously suffered retrograde and anterograde memory deficits with confabulations, severe temporal and spatial disorientation, verbal desinhibition, distrusting and suspicious behaviour, paranoia, and anosognosia. LC’s condition was attributed the diagnosis of post-TBI psychotic disorder. Neuropsychological evaluation carried out on days 87 and 89 post-injury showed severe dysfunctions in all cognitive domains (see Table 1).

**Table 1:** Scores on neuropsychological tests carried out 87 and 89 days post-injury (second hospital stay)

Tests	
Mini-Mental State Examination	17^b^
Boston Naming Test (abbreviated form of 30 items)	3^b^
Semantic verbal fluency (*Animal*s *90* s)	
- total (errors)	13 (9)^b^
Phonological verbal fluency (*P & F* 90 s)	
- total (errors)	8 (8)^b^
Category switching verbal fluency D-KEFS	
- total (errors)	0 (2)^b^
Writing to dictation	Dysorthographia
Clock Drawing (Rouleau scoring system)	6/10 Conceptual deficits and planning difficulties
Copy of the House	Normal
Mesulam Cancellation task	
- time in s	123^b^
Trail making test	
- part A (time in s)	78^b^
- part B (time in s)	215^b^
Mental Control WMS-III	20^a^
Longest Digit span forward WMS-IV	4^a^
Longest Digit span backward WMS-IV	3^a^
Logical memory (first story) WMS-IV	
- immediate free recall	3^b^
- delayed free recall	0^b^
Hopkins verbal learning test	
- total immediate free recall	13^b^
- delayed free recall	0^b^
Victoria Stroop test – interference	
- time in s	51^b^
- errors	5^b^
Matrix Reasoning WAIS-IV	12^a^
Key Search BADS	9

*BADS* behavioural assessment of the dysexecutive syndrome, *D-KEFS* Delis–Kaplan executive function system, *WAIS* Wechsler adult intelligence scale, *WMS* Wechsler memory scale

^a^ ≥ 1 ≤ 2 standard deviations away from expected mean for age and/or years of education and/or sex, according to the standards of each test

^b^ > 2 standard deviations away from expected mean for age and/or years of education and/or sex, according to the standards of each test

**Fig. 2:**
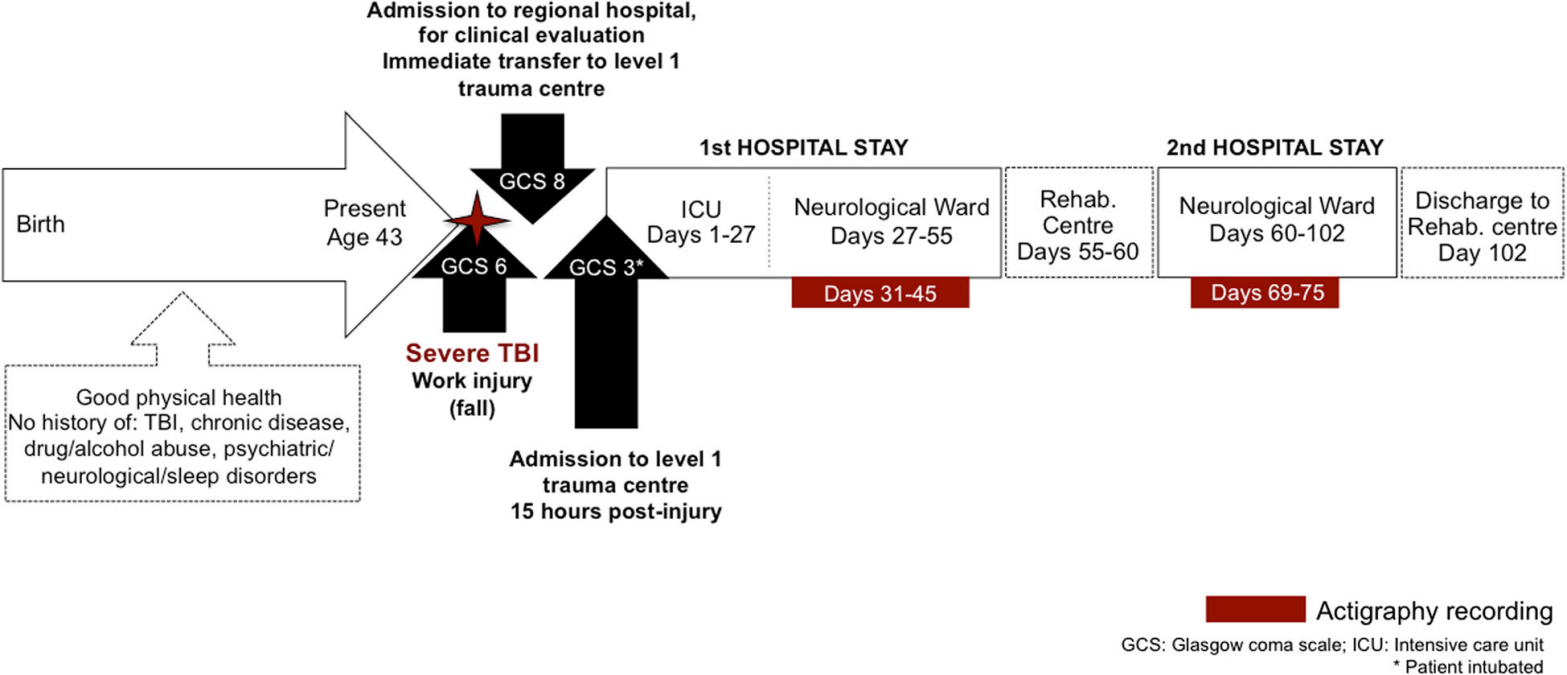
Timeline of injury, hospital stays and actigraphy. Timeline of relevant injury information, admissions and transfers, detailing the first and second hospital stays in the level I trauma centre, during which actigraphy monitoring took place

LC was discharged 43 days after this second admission (102 days post-injury), and was readmitted to the inpatient rehabilitation centre. The occupational therapy report from LC’s final evaluation, carried out one week prior to this second discharge, described him as completely dependent for domestic activities of daily living (for timeline of injury and hospital stays, see Fig. 2).

Given his lengthy second admission, his psychiatric complications and persistent cognitive and functional sequelea, LC’s case does not represent one of typical post-TBI recovery, but rather depicts a slower and complexified recovery process.

### Methods

#### Actigraphy protocol

LC was recruited as part of a larger longitudinal study taking place at *Hôpital du Sacré-Coeur de Montréal*, which was approved by the hospital ethics committee. Consent for participation was obtained from LC’s spouse, since he was unable to provide informed consent on his own.

LC wore a wrist actigraph on his non-dominant (left) arm during his first and second hospital stays (Actiwatch-2 during the first stay, and Actiwatch Spectrum during the second stay; MiniMitter Philips Healthcare, Andover, MA, USA). The actigraph is a small, watch-like device that contains an accelerometer, which records physical motion in all directions with a sensitivity of 0.05 g. Motion is then converted to an electric signal, which is digitally integrated to derive an activity count per one-min epoch. During the first hospital stay, the actigraphy recording began 31 days following the injury, 4 days after discharge from the ICU. Continuous intravenous or subcutaneous administration of a sedative drug was ceased 11 days prior to the start of actigraphic recording. LC was no longer intubated and had reached a level of medical stability defined by the absence of elevated intracranial pressure, of hemodynamic instability, and of fever or active infections. When the actigraph was installed, LC had also reached a score of IV on the Rancho Los Amigos Levels of Cognitive Functioning Scale, indicative of a confused/agitated state [13]. LC could follow simple commands for motor action inconsistently and with delay, would turn his head when his name was called. Data was acquired for 15 days during hospitalization in the regular unit, during which time he received no sedatives or analgesics. Approximately every three days, data were uploaded into dedicated software (Actiware 5.0).

During the second hospital stay, LC wore the actigraph for 7 days, beginning 69 days post-injury. During this recording period, LC received a daily dose of 3 mg of lorazepam (1 mg at 8:30 h, 17:00 h, and 22:00 h).

#### Data analyses

For each day the actigraph was worn, each minute of recording was scored as “sleep” or “wake” using the automatic scoring system of the dedicated software (Actiware 5.0). A particular one-min epoch was scored as wake by comparing the activity counts of this epoch to those immediately surrounding it. The threshold chosen to score a one-min epoch as wake was > 20 activity counts per minute. A smaller number yielded a score of sleep. For each epoch the actigraph was not worn, due to the removal of the actigraph for data downloads or bathing, the epoch was scored as wake since the patient was awake in both contexts.

A sleep bout was defined as a period of five or more consecutive epochs scored as sleep by Actiware 5.0. To reduce the artificial fragmentation of rest periods, isolated one-min epochs scored as wake were manually converted to sleep, similar to the smoothing method suggested by Sitnick et al. [14].

Sleep efficiency was calculated for the nocturnal period (22:00 h to 6:59 h), and was defined as [(number of epochs scored as sleep / total number of nocturnal epochs)*100].

Sleep-wake cycle consolidation, or the clustering of activity during the daytime and of rest during the nighttime, was estimated with the ratio of daytime activity to total 24-h activity, as previously described [7]. Briefly, for each 24-h period, the activity counts were summed separately for daytime (07:00 h −21:59 h) and nighttime (22:00 h - 6:59 h) periods. Total 24-h activity (07:00 h - 06:59 h) was the sum of the daytime and nighttime periods. The percentage of total 24-h activity occurring in the daytime was calculated to obtain the daytime activity ratio [daytime activity ratio = (daytime activity/24-h activity) x100].

#### Statistical analyses

Descriptive statistics (means and standard deviations) were computed for the total quantity of sleep per 24-h period, mean duration of daytime and nighttime sleep bouts (“sleep bout duration”), the nocturnal sleep efficiency, and the daytime activity ratio. Student’s t-tests were carried out to assess differences in these results between the first and second hospital stays.

### Results

**Fig. 3:**
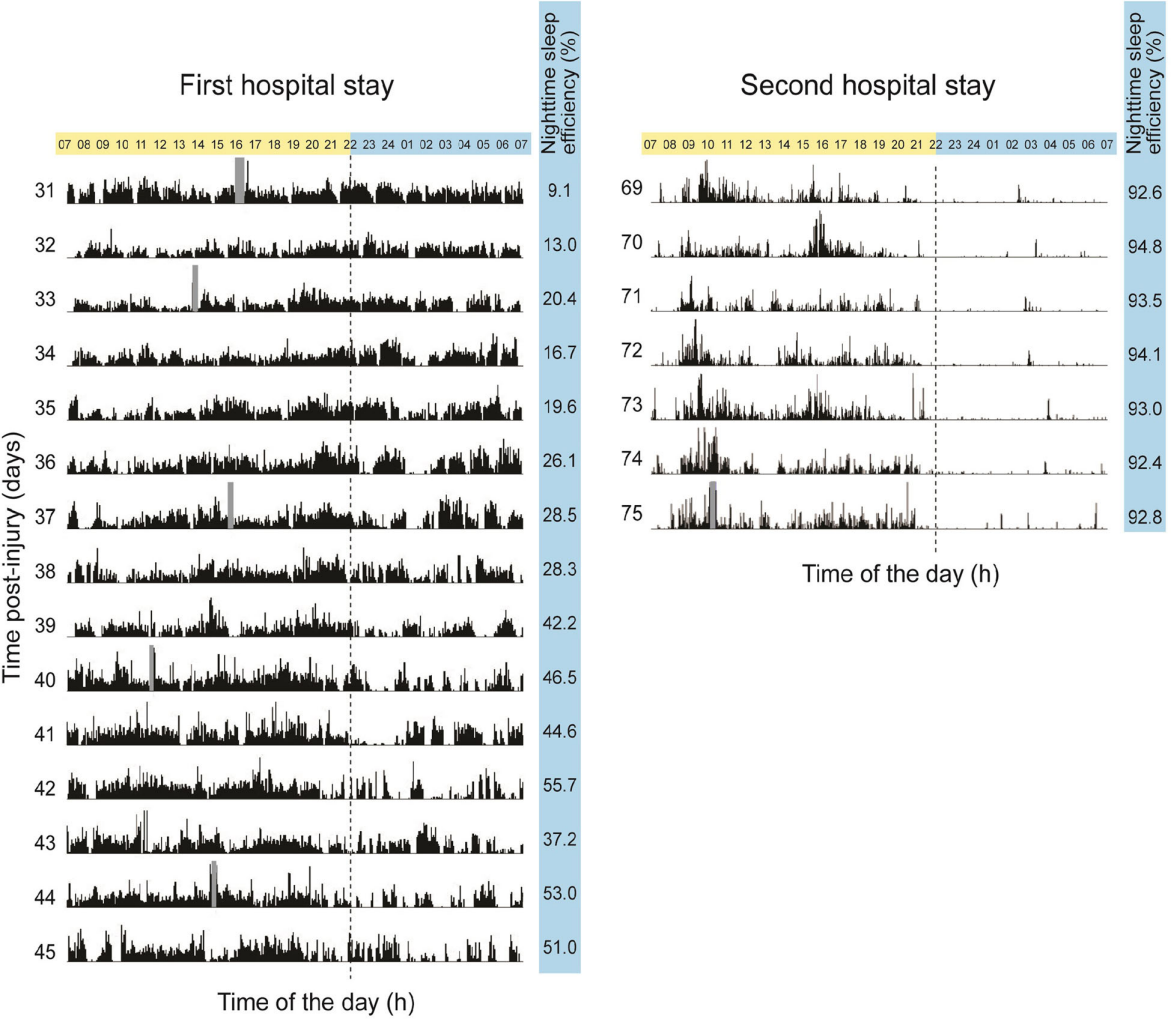
Actigraphy recordings of the first and second hospital stays. Each of the 15 and 7 days of recording are represented on a separate line, from 07:00 to 07:00 h. Total activity counts for each minute of recording is illustrated by vertical dark lines. The same scale of 0 to 1000 activity counts was used for all days of both hospital stays. Hours included in the day period (07:00 to 22:00 h) are shown in yellow and those included in the night period (22:00 to 07:00 h) are in blue at the top of each graph. The number on the left of each day of recording corresponds to the day post-injury. Nocturnal sleep efficiency is indicated on the right column of each actigram

The actigraphy recordings for the first and second hospital stays are presented in Fig. 3. During the first hospital stay, high levels of activity were dispersed throughout 24-h periods for most of the 15 days of recording, and very brief periods of sleep are observed. As for the second hospital stay, prolonged periods of sleep are observed, mostly during nighttime.

### Total quantity of sleep per 24-h period

During the actigraphy recording of the first hospital stay, LC had an average of 4.8 ± 1.3 h of sleep per 24-h period, which significantly increased to 14.1 ± 0.9 h during the second hospital stay (t(20) = −16.8, *p* < 0.001) (see Fig. 3).

### Duration of sleep bouts

During the first hospital stay, sleep bouts had an average duration of 14.9 ± 11.9 min and the longest sleep bout over the 15 days of actigraphy was 97 min (occurring at 22:39 h on day 41 post-injury). During the second stay, sleep bouts were on average 38.4 ± 59.0 min, which represents a significant improvement compared to the first hospital stay (t(400) = −6.2, *p* < 0.001). The longest sleep bout started at 21:16 h on day 72 post-injury and was of 342 min in duration. During the night, averaged sleep bout was significantly longer during the second hospital stay, increasing from 16.6 ± 13.7 min in the first stay to 90.1 ± 88.6 min in the second stay (t(192) = −9.9, *p* < 0.001). The average duration of daytime sleep bouts also increased from the first to the second hospital stay, from 12.5 ± 8.3 min to 17.9 ± 17.3 min (t(206) = −2.9, p = 0.005).

### Sleep efficiency

Nocturnal sleep efficiency increased significantly from the first to the second hospital stay (32.7 ± 15.4 % vs. 96.3 ± 0.9 %, t(20) = −10.27, *p* < 0.001).

### Rest-activity cycle consolidation

When all days of recording were considered for each hospital stay, daytime activity ratio was 67.8 ± 9.8 % during the first hospital stay, and 96.2 ± 1.0 % during the second hospital stay, which represents a significant improvement in sleep-wake cycle consolidation (t20) = −7.53, *p* < 0.001)).

## Conclusions

We presented the case of a 43-year old male, who suffered significant sleep-wake disturbances in the first 3 months post-TBI. LC’s first hospital stay was marked by an average of only 4.8 ± 1.3 h of sleep per 24-h for the 15 days of recording. Importantly, this short sleep duration measured with actigraphy probably overestimates the quantity of sleep LC actually experienced. In fact, actigraphy is known to underestimate wakefulness [15–18], particularly when individuals lie in bed immobile but awake [19], and especially among a critically ill population [20]. On the other hand, it is not impossible that LC may have slept during periods of motor activity. However, the recorded levels of activity were very high (see Fig. 3), suggesting that if sleep did occur, it was agitated and most likely not restful. Taken together, our results suggest severe and persistent sleep deprivation during the first hospital stay.

Aside from sleep deprivation, this study also suggests that LC suffered severe sleep fragmentation. The patient was not able to stay asleep for a long period of time (mean nighttime sleep bout duration of 16.6 ± 13.7 min), and the mean sleep efficiency of 32.7 % measured during the first hospital stay was well below the 85 % mark that is generally considered pathological [21]. Altogether, these results demonstrate that sleep was highly disturbed during the first hospital stay. Such a pattern of sleep is most likely incompatible with the deeper sleep stages associated with recovery, although this cannot be confirmed with actigraphy measures alone.

When LC’s sleep-wake cycle was re-evaluated during the second hospital stay, LC was able to have significantly longer periods of continuous bouts of sleep, especially during the night. Sleep efficiency improved significantly, increasing from 32.7 ± 15.4 % to 96.3 ± 0.9 %. Total quantity of sleep per 24-h period also increased from 4.8 ± 1.3 h during the first stay to 14.1 ± 0.9 h during the second stay. Moreover, periods of activity were mainly concentrated during the daytime (daytime activity ratio of 96.2 ± 1.0 %), suggesting the presence of a well-consolidated sleep-wake cycle. During the second hospital stay, LC’s sleep pattern may be more closely aligned with hypersomnia, which is reported in approximately 10-30 % of TBI patients in the post-acute and chronic phases of injury [1, 22].

During the actigraphy recording of his first hospital stay, LC was hospitalized in the neurological ward, in a room of 6 patients, and was re-hospitalized in the same ward during his second hospital stay, in a two-patient room. In this ward, hallway lights are generally turned on from 7:00 h to 22:00 h, and the hospital personnel attempts to keep noise and light levels as low as possible between 22:00 h and 7:00 h. Considering the significant improvement in sleep-wake cycle consolidation during the second hospital stay, despite LC being hospitalized in the same ward, this case study suggests that the hospital environment cannot entirely account for the sleep deprivation and sleep disturbances occurring in patients with TBI.

Being under the effects of sedatives, analgesics, narcotics, anticonvulsants and antipsychotics may also influence sleep characteristics during acute hospitalization following TBI [23]. Furthermore, withdrawal from such medications may also influence sleep and wake. As LC was discharged from the ICU only 4 days prior to the start of actigraphy, the sleep-wake cycle measured during his first hospital stay may have been influenced by withdrawal from the sedatives and analgesics administered while he was in the ICU. Conversely, improvements in sleep-wake cycle consolidation during the second hospital stay, including longer nighttime sleep periods, could partially be due to the effect of lorazepam, as LC was not taking analgosedative medication during the 15 days of actigraphy recording of his first stay. However, since equal (1 mg) doses were administered three times daily (8:30 h, 17:00 h, 22:00 h) during the second stay, and not exclusively prior to bedtime, LC’s consolidated daytime wakefulness and nighttime sleep cannot be due solely to the effect of medication.

Pain may also be an important contributing factor to sleep disturbances following TBI. LC had multiple fractures, which most likely generated significant pain. In the chronic phase of TBI (all severities), pain is known to negatively influence sleep [24–27], as early as the post-acute period [28]. Among ICU patients without TBI, pain has also been associated to sleep disturbances [29–31]. The influence of pain on LC’s sleep may have been stronger during the first hospital stay, as pain may have gradually subsided with time, though no pain evaluations were systematically carried out due to akinetic mutism.

### Clinical implications

This case report is the first to extensively document sleep-wake disturbances in both the acute and subacute phases of severe TBI. Indeed, this was the only case we encountered of a patient being readmitted shortly after discharge, providing us with a unique opportunity to follow-up on our actigraphy measures. This successive monitoring of LC’s sleep-wake cycle, while in the same hospital ward, distinguishes the present study from previously published TBI sleep studies [2, 3, 5, 6], including our own [7]. Results revealed the presence of severe sleep deprivation and the absence of normal 24-h sleep-wake organisation during the acute phase after a severe TBI. Severe sleep deprivation is bound to have negative consequences on physical, psychological and cognitive recovery following TBI. Indeed, post-TBI sleep disturbances have been shown to heighten cognitive, mood and communication impairments, in addition to intensifying pain and compromising recovery [32, 33]. In a more general manner, partial or chronic sleep deprivation has been shown to negatively impact cognitive, behavioural, immune, inflammatory, cardiovascular, endocrine and metabolic functions [34–38]. In the case of LC, severe and persistent sleep deprivation and fragmentation, as well as the severe disturbance of the sleep-wake cycle in the first hospital stay, may have contributed to the psychiatric condition having led to his second hospital admission. Indeed, sleep and circadian disturbances are associated to mental health and psychiatric symptoms and disorders [39–41], while sleep deprivation has been associated with psychotic symptomatology [42].

The sleep deprivation experienced by LC was much more severe and prolonged and than that of other moderate-severe TBI evaluated within our larger study [7]. Interestingly, the case of LC differs from previously observed cases of TBI patients for whom improved sleep and wake seem to coincide with improved cognitive functions in the weeks following injury [5, 7, 43] Rather, LC had persistent PTA, cognitive deficits and psychiatric symptoms, despite significant improvement of sleep-wake cycle consolidation from the first to the second hospital stay. This may suggest that severe and prolonged sleep deprivation in acute TBI could possibly exacerbate cerebral damage and have persistent effects on cognitive sequelea and recovery.

No sleep medication was given to LC during the actigraphy recording period of the first hospital stay, during which he was suffering from severe sleep deprivation, probably because he was not able to communicate his sleep problem. Systematic monitoring of sleep by observation are difficult to conduct and quite time-consuming. It is therefore rarely included in the nursing care, especially in patients in such severe medical conditions. Actigraphy may be particularly useful among patients with confusion or communication deficits, as it objectively identifies sleep patterns and may contribute to providing timely and adequate treatment if sleep disturbances arise. The sleep disturbances experienced by LC could probably have been attenuated, though the means through which sleep can be facilitated within this population still need to be further investigated.

This report highlights the importance of monitoring the sleep-wake cycle in acute care, as it may inform or influence patient recovery, though more studies are needed to define this relationship and determine whether it is causal or bidirectional. Even though actigraphy cannot distinguish rest from sleep, it remains a useful tool for the prolonged measurement of sleep-wake disturbances in a hospital setting, even among patients who may lack the cognitive capacity to identify and/or report sleep-wake problems to healthcare personnel.

### Limitations

One limitation to this study is that no magnetic resonance imaging was performed. Given its superior spatial resolution compared with CT [44], magnetic resonance imaging would have enabled a more precise detection of alterations in cortical and subcortical structures and networks involved in the regulation of sleep and wake. However, with the CT scan at admission, we were still able to detect petechiae within the pons, which is a region highly involved in sleep-wake regulation [45, 46].

## Additional files

Additional file 1: Raw actigraphy data of the first hospital stay, comprising activity counts and sleep/wake scoring per one-min epoch. (XLS 1734 kb) Additional file 2: Raw actigraphy data of the second hospital stay, comprising activity counts and sleep/wake scoring per one-min epoch. (XLSX 348 kb)

## Abbreviations

CT: Computed tomography
GCS: Glasgow coma scale
ICU: Intensive care unit
PTA: Posttraumatic amnesia
TBI: Traumatic brain injury
